# Neuropsychological assessment in adult epilepsy: rationale, test selection, and clinical integration across non-surgical care in Italian patients

**DOI:** 10.1007/s10072-026-09174-2

**Published:** 2026-06-29

**Authors:** Francesco Tomaiuolo, Carmelo Mario Vicario, Davide Cardile, Serena Campana, Rocco Salvatore Calabrò, Francesco Corallo, Angelo Labate

**Affiliations:** 1 Department of Clinical and Experimental Medicine, University Hospital of Messina, University of Messina, Messina, 98125 Italy; 2 Department of Cognitive Sciences, Psychology, Education and Cultural Studies, University of Messina, Messina, 98125 Italy; 3 IRCCS Centro Neurolesi “Bonino-Pulejo”, Messina, Italy; 4 Neurorehabilitation Unit, Auxilium Vitae Volterra, Via Borgo San Lazzero 5, Volterra, 56048 Italy; 5 Neurology Clinic, University of Palermo, Palermo, Italy; 6 Department of Biomedicine, Neuroscience, and Advanced Diagnostic (BIND), University of Palermo, Palermo, Italy

**Keywords:** Epilepsy, Neuropsychological assessment, Cognition, Clinical management, Adult epilepsy, Patient-reported outcomes

## Abstract

To provide a practical framework for neuropsychological assessment in adult epilepsy, specifying what each measure evaluates and how results can support clinical decisions regarding epileptic patients. This work is a clinically oriented narrative review and expert proposal for non-surgical adult epilepsy care in Italian settings. Test selection was guided by domains repeatedly emphasized in epilepsy neuropsychology, by their practical relevance to routine clinical decision-making, by compatibility with major international recommendations, and by the availability of Italian adaptations or normative data. For each test, we describe the primary construct assessed and the specific epilepsy-related clinical question it may help address. Structured cognitive exams can be sensitive to lateralization and localization, medication-related cognitive effects, and comorbid mood and anxiety symptoms. When integrated with electroencephalography and structural imaging, these profiles support clinical interpretation and guide counseling. Serial assessment can also inform medical management, rehabilitation planning, and quality-of-life interventions. The expanded framework clarifies which patients should be referred for assessment, why epilepsy must be conceptualized as a heterogeneous group of syndromes and network disorders, where neuropsychological data may support but not replace differential diagnosis, and how current ILAE recommendations contextualize the proposed battery. Neuropsychological assessment is a core component of comprehensive epilepsy care, but its clinical value is greatest when the battery is tailored to the referral question, syndrome, disease stage, treatment context, and patient priorities. The proposed battery should be read as a structured, adaptable framework for routine adult non-surgical practice, rather than as a rigid battery to be applied uniformly across patients.

## Introduction

Epilepsy is a chronic brain disorder characterized by recurrent unprovoked seizures and a broad range of associated cognitive, psychological, and social consequences [1]. In adults, difficulties in memory, language, attention, processing speed, and executive functions are frequent and clinically meaningful. These difficulties may reflect the underlying epileptogenic pathology, seizure burden, disease duration, antiseizure medication effects, network reorganization, and psychosocial factors [2, 3].

Neuropsychological assessment serves to characterize the individual cognitive and behavioral profile and to support management, including education and counseling, rehabilitation planning, work and driving recommendations, and treatment choices when cognition is a concern. In routine care, referral is particularly appropriate when patients report persistent cognitive complaints, show functional decline in everyday life or work, present with suspected medication-related adverse cognitive effects, have psychiatric comorbidity that may complicate interpretation of symptoms, or require a baseline for future comparison after major therapeutic changes [4–6]. Referral is also appropriate when the neurologist needs a more formal estimate of strengths and weaknesses to contextualize electroclinical and imaging data, or when diagnostic uncertainty persists despite standard work-up. For patients who are candidates for surgery, neuropsychological assessment is part of the broader multidisciplinary evaluation, but the present article focuses on non-surgical settings [5, 7].

Our aim is to respond to the operational needs of Italian epilepsy services. Accordingly, test selection and sequencing prioritize instruments with established clinical utility and, whenever possible, Italian adaptations or normative data. Because assessment results can influence major clinical decisions, we assume administration and interpretation by clinicians with formal training in neuropsychology, using standardized procedures and careful qualitative observation. Methodologically, this manuscript should be regarded as a clinically oriented narrative review and expert proposal. It was not developed through a formal Delphi process, nor through a systematic or scoping review with predefined search and selection procedures. We now make this premise explicit because the intended contribution of the paper is pragmatic: to organize a feasible battery for everyday adult epilepsy care in Italy while keeping it aligned with the conceptual framework and minimum standards proposed by international epilepsy neuropsychology groups, particularly the International League Against Epilepsy (ILAE) recommendations for routine care [5].

The proposed battery of tests is organized to reduce diagnostic ambiguity and to support efficient clinical integration. We begin with basic abilities (sensory-motor skills, basic visuo-perceptual processing, visuo-construction, and core language) to avoid misattributing failures in higher cognitive processing to upstream sensory-motor or perceptual constraints. We then evaluate attention and processing speed, learning and memory (with attention to material-specific patterns), executive control, and patient-reported outcomes. Importantly, this sequence should not be interpreted as implying that all adults with epilepsy require an identical full battery. Rather, the battery is modular: some patients need broad baseline characterization, whereas others require targeted assessment driven by a specific question such as medication tolerability, return to work, differential diagnosis, or longitudinal monitoring after a clinically meaningful change. This flexibility is especially important because epilepsy is not a homogeneous disease, but a group of syndromes and etiologies that differ in age at onset, seizure type, localization, network involvement, lesion burden, psychiatric comorbidity, and expected cognitive phenotype [2–4, 8]. The same verbal memory test, for example, may carry different interpretive weight in mesial temporal lobe epilepsy, generalized epilepsies with prominent attentional inefficiency, autoimmune or inflammatory epilepsies, developmental and genetic epilepsies, and functional events misdiagnosed as epilepsy [9, 10]. Neuropsychological testing therefore supports interpretation alongside electroencephalography and neuroimaging, and it facilitates communication with the multidisciplinary epilepsy team.

## Methods

### Study design

This article is a clinically oriented narrative review and expert proposal. It was not developed through a formal Delphi consensus process, nor through a systematic or scoping review with predefined eligibility criteria, PRISMA-guided screening, or quantitative synthesis. This methodological choice was deliberate: the primary aim was not to establish the superiority of one test over another through meta-analytic evidence, but to organize a feasible, clinically interpretable, and locally applicable neuropsychological battery for routine adult epilepsy care in non-surgical Italian settings. The framing as a narrative review is consistent with the exploratory and pragmatic nature of the contribution, and with the current state of the literature, which does not yet support a single evidence-based universal battery for this population.

### Literature search strategy

A narrative search of the scientific literature was conducted using PubMed, PsycINFO, and Google Scholar, without date restrictions, with a final update in January 2026. Search terms included combinations of the following keywords: epilepsy, neuropsychological assessment, cognitive assessment, adult epilepsy, neuropsychology, cognition, antiseizure medication, cognitive side effects, quality of life, epilepsy neuropsychology battery, ILAE recommendations, temporal lobe epilepsy, psychogenic nonepileptic seizures, and Italian normative data. Additional references were identified through manual screening of the reference lists of key retrieved articles, consensus documents, and ILAE Task Force reports. Priority was given to peer-reviewed empirical studies, standardization and normative studies, clinical guidelines, and systematic reviews or meta-analyses where available.

### Test selection criteria

Individual neuropsychological instruments were selected according to the following pre-specified criteria, applied jointly:

- *Domain relevance*: the instrument must assess a cognitive or behavioral domain repeatedly identified as clinically meaningful in adult epilepsy neuropsychology, as documented in the empirical literature and in major international guidelines [4, 5].
- *Clinical utility*: the instrument must address a specific and actionable clinical question in routine non-surgical epilepsy care (e.g., lateralization, medication tolerability monitoring, differential diagnosis, rehabilitation planning, or quality-of-life evaluation).
- *Psychometric adequacy*: the instrument must have documented reliability and validity for the intended purpose, based on published standardization or normative studies.
- *Local applicability*: wherever possible, instruments with published Italian normative data or validated Italian adaptations were preferred, to ensure that individual scores can be meaningfully interpreted against an appropriate reference population.
- *Feasibility*: the overall battery was designed to be administrable within routine outpatient epilepsy services, balancing comprehensiveness with time and resource constraints typical of Italian clinical settings.

Instruments that lacked Italian normative data were included when no comparable validated alternative existed for the relevant domain, and when their clinical utility was considered to outweigh the interpretive limitations. In such cases, this limitation is explicitly noted in the corresponding section.

### Scope and limitations

This proposal covers adult patients (18 years and older) with epilepsy who are managed in non-surgical settings. Pediatric epilepsy, presurgical evaluation, and intraoperative neuropsychological assessment are explicitly excluded from the scope of the present framework, as they require distinct methodological and normative considerations. The proposed battery should be regarded as a structured and adaptable clinical framework rather than as an empirically validated universal protocol. Controlled studies directly comparing battery configurations, referral strategies, and longitudinal outcomes in defined adult epilepsy populations are needed to provide a stronger evidence base for future recommendations.

## Test battery overview: constructs and epilepsy-specific clinical questions

### Sensory-motor foundations

These measures provide anchors for interpreting timing performance, left/right asymmetries, and global psychomotor slowing which are frequently influenced by seizures and antiseizure medications in people with epilepsy (e.g., slowed motor speed, reduced coordination) [11].

- **Nine Hole Peg Test**: fine motor dexterity and visuo-motor coordination.

The performance helps contextualize speeded measures and can highlight lateralized motor slowing related to focal pathology or asymmetric treatment effects, supporting differential interpretation when a suspected lesion or seizure focus involves the motor network [12]. Subtle slowing in manual dexterity is clinically important because psychomotor speed and hand-eye coordination are often compromised by both seizures and antiseizure polytherapy, with downstream impact on driving, employment and everyday independence [13].

### Language

Language testing informs overall functional impact, supports lateralization when interpreted cautiously, and helps contextualize language functioning in selected cases, especially in dominant-hemisphere temporal lobe epilepsy. Naming and word-finding difficulties are commonly reported and can significantly interfere with everyday communication and quality of life [14–16].

- **Esame Neuropsicologico per l’Afasia (** *ENPA* - [17]): lexical retrieval and visual confrontation naming.

The naming performance is sensitive to dominant-hemisphere temporal dysfunction and provides a clinically interpretable baseline for counseling and follow-up before and after surgery. Persistent anomia is one of the most frequent and disabling language complaints in left temporal lobe epilepsy, with significant everyday impact on social participation and subjective well‑being [18].

- **Token Test**: auditory comprehension under increasing syntactic and working memory demands.

This measure helps detect subtle receptive language inefficiency and can help interpret performance on verbally mediated tasks [19, 20]. In focal epilepsies involving temporo‑parietal networks, mild comprehension deficits may exacerbate functional difficulties at work and in complex daily communication even when basic conversation appears intact [14].

- **Verbal Fluency** (*phonemic and semantic*): rapid word generation under phonemic constraints and semantic category retrieval. Fluency tasks capture initiation speed, retrieval efficiency, and executive-linguistic control; they are informative in fronto-temporal network vulnerability and medication-related slowing [21, 22]. Executive‑language inefficiency, including slowed and effortful fluency, has been linked to poorer psychosocial functioning and lower health‑related quality of life in people with epilepsy, particularly when executive dysfunction is prominent [23, 24].

### Visuo-perceptual and Visuo-spatial Construction

These tasks help characterize posterior network functioning and provide a baseline for interpreting non-verbal learning and memory. Visual-spatial deficits are reported across several epilepsy syndromes and may be particularly relevant in right temporal and parietal epilepsies, where they can compromise driving, navigation and complex visuospatial activities [25].

- **Judgment of Line Orientation**: visuospatial judgment of angular orientation with minimal motor requirements. This measure is useful for detecting visuo-spatial inefficiency and for interpreting non-verbal memory measures that rely on accurate perception [26–29]. Visuospatial difficulties, especially in patients with right temporal involvement, can contribute to problems with orientation, reading maps and handling spatially complex work tasks, thereby affecting functional independence and quality of life [30].
- **Rey-Osterrieth Complex Figure**, *Copy*: visuo-construction, organization, and perceptual integration.

Copy performance establishes the quality of encoding material for later recall; disorganization can reflect posterior dysfunction or executive planning difficulties [31–34]. In temporal lobe epilepsies, visual-spatial aspects of the Rey-Osterrieth test can help characterize lateralized deficits and have been used to document non‑verbal memory impairment and accelerated long‑term forgetting, which are frequent, functionally relevant complaints in both temporal and generalized epilepsies [35, 36].

- **Block Design** (*in WAIS-IV*): timed visuo-spatial analysis and construction.

Block Design provides a standardized index of visuo-spatial problem solving with a speed component that can be sensitive to generalized cognitive slowing [37, 38]. Reduced performance can signal vulnerabilities in spatial reasoning and processing speed that are particularly important for occupations involving complex visuospatial demands and rapid visual integration [39].

### Attention, Processing Speed and Short-Term Memory

Speed and attentional efficiency are among the most commonly affected in epilepsy and are particularly important for longitudinal monitoring and functional counseling. Across syndromes, 30–40% of adults with chronic epilepsy show measurable cognitive impairment, with attention, reaction speed and executive functions often most affected and sensitive to both seizure burden and antiseizure medication effects [39, 40].

- **Digit Span** (*forward/backward/sequencing*): auditory attention and short-term memory/working memory manipulation.

Digit Span anchors interpretation of learning and executive tasks and can reflect diffuse network inefficiency or medication burden [41]. Working‑memory difficulties have been associated with transient epileptiform discharges during testing and with higher lifetime seizure burden, highlighting the importance of monitoring these functions when counseling patients about work capacity and academic performance [30, 42].

- **Symbol Digit Modalities Test**: processing speed and divided visual attention.

This test is highly sensitive to generalized slowing; it is useful for serial monitoring across changes in seizure control or medication regimen [37, 43]. Attention and processing speed are repeatedly identified as key cognitive vulnerabilities in epilepsy and may partially improve with targeted pharmacological or behavioral interventions. Slowed processing speed is clinically salient because it predicts poorer academic and occupational functioning and can reduce self‑efficacy in disease self‑management. [44, 45].

- **Trail Making Test A** *(TMT-A*): visual scanning and psychomotor speed.

*TMT*-A provides a baseline for interpreting set shifting on Trail Making Test B and helps quantify global speed changes under visual search demands [46–48]. Psychomotor slowing is a frequent complaint in adults with epilepsy and has been linked to higher antiseizure drug load and greater seizure burden, both of which are associated with poorer everyday functioning and reduced quality of life [13].

- **Trail Making Test B** *(TMT-B*): set shifting and mental flexibility under time pressure.

TMT-B is sensitive to inefficiency of executive processing and can capture the functional cost of switching and divided attention [46–48]. Executive dysfunction, including impaired set‑shifting, has been shown to predict lower health‑related quality of life in pediatric and adult epilepsy populations, over and above some traditional neurological variables [24, 49, 50].

### Learning and Memory

Memory assessment should consider both verbal and non-verbal materials, encoding quality, interference effects, and retrieval versus recognition processes. Episodic memory difficulties are highly prevalent in temporal lobe epilepsies and are also reported in generalized epilepsies, often contributing more to functional disability than seizure frequency itself [36].

- **List learning**: Rey Auditory Verbal Learning Test (Rey’s 15-Word Test) learning across trials, retention, interference susceptibility, and recognition.

List learning characterizes verbal episodic learning and supports counseling about everyday memory; characteristics patterns may be informative in temporal lobe syndromes [51–54]. Verbal learning deficits and accelerated long‑term forgetting have been documented in both temporal and generalized epilepsies and are frequently associated with subjective memory complaints and difficulties in education and work, underlining their importance for treatment planning [30, 55].

- **Story recall**: contextual verbal episodic memory and narrative organization.

This test complements list learning when strategy use or fluency may confound performance and can clarify the functional impact of verbal memory difficulties (in Italian there are three parallel versions: [32, 56, 57]). Narrative memory is clinically meaningful because patients often report difficulties retaining conversations, instructions and everyday events, which can contribute to perceived unreliability and stigma in social and occupational settings [58, 59].

- **Rey-Osterrieth Complex Figure** (*Immediate and Delayed Recall*): non-verbal learning and retrieval of complex visuo-spatial material.

Delayed recall helps differentiate non-verbal memory impairment from visuo-constructional or organizational weaknesses observed on the condition involving the Rey-Osterrieth Figure [31, 32, 34]. Visual memory impairments and accelerated forgetting on the Rey-Osterrieth test have been reported in both temporal lobe and generalized genetic epilepsies and are associated with broader cognitive decline and everyday memory complaints [35, 36, 55].

- **Matched recall and recognition across modalities** (e.g., Doors and People or equivalent): recall and recognition with comparable task demands for verbal and visual material.

These paradigms support interpretation of whether the impairment reflects encoding, storage, or retrieval limitations and improves cross-domain comparability [60, 61]. Distinguishing retrieval versus storage deficits is crucial for prognosis and for counseling about compensatory strategies, as storage‑level problems tend to be more resistant to cueing and to negatively influence long‑term independence [30, 39]

### Executive functions and cognitive control

Executive measures capture inhibitory control, cognitive flexibility, abstraction, and error monitoring, which are important for self-management and daily functioning. Executive dysfunction is common in epilepsy and has been linked to difficulties with treatment adherence, driving, employment and social participation, as well as poorer health‑related quality of life [50].

- **Stroop Color-Word** (*or equivalent interference task*): inhibitory control and resistance to interference. Stroop-type task are relevant to everyday self-regulation and can be sensitive to diffuse executive vulnerability and medication effects [62]. Impaired inhibition may translate into difficulties suppressing responses, managing emotional reactivity, and resisting distractions, which can undermine self‑management behaviors such as medication adherence and lifestyle modification [63].
- **Modified Card Sorting Test**: abstraction, set shifting, and perseveration.

Performance on card sorting tasks evaluates flexible problem solving and error monitoring, especially when executive limitations affect work and social functioning [64, 65]. Studies have shown that executive dysfunction contributes independently to poorer academic and occupational outcomes and is associated with lower quality of life, even after accounting for seizure variables [24, 49, 50].

### Patient-reported outcomes

Brief standardized symptom and quality-of-life measures provide essential context for interpreting cognitive findings and for tracking outcomes meaningful to patients. Psychiatric comorbidities and reduced health‑related quality of life are highly prevalent in adults with epilepsy and often exert a stronger impact on subjective well‑being than seizure frequency alone [66, 67].

- **Depression inventory** (e.g.,* BDI-II or validated equivalent*): depressive symptom severity.

Screening supports identification of treatable comorbidity that can worsen subjective cognitive complaints and reduce environmental adaptation [68, 69]. Numerous studies using BDI‑II or epilepsy‑specific depression scales show that depressive symptoms are among the strongest predictors of lower QOLIE‑31 scores, often explaining more variance in quality of life than seizure frequency or seizure type [70, 71].

- **Anxiety screening**: anxiety symptom burden (e.g.,* GAD-7 or Beck Anxiety Inventory*).

Anxiety is a frequent comorbidity that can affect attention, sleep, and perceived cognitive function and is associated with poorer quality of life in both focal and generalized epilepsies [72–74]. Even mild anxiety and depressive symptoms have been shown to significantly reduce quality of life scores, underscoring the importance of systematic screening and integrated treatment in epilepsy care [66, 75].

- **Epilepsy-specific quality of life measure** (e.g.,* QOLIE-31 or validated equivalent*): multi-domain health-related quality of life, including cognition, mood, and medication effects.

QOLIE-31 and related instruments documents patient-reported change beyond seizure frequency and helps prioritize clinical interventions [76–78]. Lower QOLIE‑31 scores have been associated with higher seizure frequency, greater psychiatric burden, lower socioeconomic status and reduced employment, making these measures critical outcomes for both routine clinical monitoring and research trials [63, 66, 70].

### Clinical integration in non-surgical care

Neuropsychological results should be interpreted in the context of a convergent dataset that includes clinical history, seizure semiology, electroencephalography, neuroimaging, psychiatric status, and where relevant social and occupational functioning. In this sense, the proposed battery is not intended to generate a diagnosis in isolation, but to strengthen clinical formulation when considered together with the rest of the case.

For non-surgical care, the test battery offers actionable targets for clinical management. Processing speed and attention measures help quantify cognitive tolerability when antiseizure regimens are adjusted [6]. Memory and executive findings support individualized rehabilitation strategies, compensatory skill training, and recommendations for daily functioning. Mood and anxiety screening, together with quality-of-life measures, help distinguish primary cognitive impairment from affective contributors and guide referral for psychological or psychiatric care [4]. Neuropsychological data may also contribute to differential diagnosis, although this contribution must be described cautiously. In particular, patients with psychogenic nonepileptic seizures (PNES) or other functional disorders may present with cognitive complaints that resemble those observed in epilepsy. Neuropsychological testing can help identify patterns of inconsistent effort, pronounced affective contribution, or cognitive complaints that are disproportionate to objective findings; however, these data are adjunctive and should never be treated as stand-alone proof of PNES. Current ILAE recommendations emphasize staged diagnosis based primarily on clinical history and video-EEG evidence, while the neuropsychological examination may help characterize comorbidity, functional burden, and rehabilitation needs [9].

The suggested neuropsychological tests for the assessment of adults with epilepsy in non-surgical clinical settings are summarized in Table 1, which outlines the main cognitive domains covered by the proposed battery, the specific purpose of each test, and the key supporting references. This selection is intended as a practical framework to support baseline characterization, longitudinal follow-up, and the interpretation of cognitive complaints in routine clinical practice. The table should therefore be read as a structured aid to clinical reasoning rather than as an empirically validated universal battery. Controlled data support the clinical usefulness of neuropsychological assessment in epilepsy for identifying comorbidity, quantifying medication effects, informing counseling, and monitoring change over time, but the literature is less definitive in supporting one fixed battery for all non-surgical patients. This limitation is one reason we prefer an explicitly pragmatic formulation of the present proposal.

**Table 1:** Suggested neuropsychological tests for adults with epilepsy in non-surgical clinical settings, with the main construct assessed, clinical utility, and key references

Domain	Test	What it assesses/purpose	Key reference(s)
*Sensory-Motor Foundations*	Nine Hole Peg Test	Fine manual dexterity, visuomotor coordination, and lateralized motor slowing.	Oxford Grice et al., [12]
*Language*	ENPA	Lexical retrieval and visual naming.	Capasso and Miceli, [17]
	Token Test	Auditory comprehension with progressively increasing syntactic and working-memory demands.	Renzi and Vignolo, [19], Spinnler & Tognoni, [20]
	Verbal fluency (phonemic and semantic)	Rapid word generation, lexical retrieval efficiency, and executive-linguistic control.	Novelli et al., [21]; Capitani et al., [22]
*Visuoperceptual and Visuospatial Construction*	Judgment of Line Orientation	Visuospatial judgment of angular orientation with minimal motor demands.	Benton et al., 1992; [29]
	Rey-Osterrieth Complex Figure - Copy	Visuoconstruction, organization, and perceptual integration.	Caffarra et al., [31], Carlesimo, [32], Rey, [33], Shin, [34]
	Block Design (WAIS-IV)	Visuospatial analysis and construction with a speed component.	Wechsler, [37]; Orsini & Pezzuti, [38]
*Attention*,* Processing Speed and Short Term Memory*	Digit Span (forward/backward/sequencing)	Auditory attention and working memory.	Monaco et al., [41]
	Symbol Digit Modalities Test	Processing speed and divided visual attention.	Wechsler, [37], Smith [43]
	Trail Making Test A	Visual scanning and psychomotor speed.	Amodio et al., [46], Reitan, [47], Siciliano, [48]
	Trail Making Test B	Set shifting and mental flexibility under time pressure.	Amodio et al., [46], Reitan, [47], Siciliano, [48]
*Learning and Memory*	List learning (Rey Auditory Verbal Learning Test/Rey 15-Word Test)	Verbal learning across repeated trials, retention, interference, and recognition.	Caltagirone et al., [54], Carlesimo et al.,[51–53]
	Story recall	Contextual verbal episodic memory and narrative organization.	Carlesimo et al., [32], Mondini, [57], Mondini, [56]
	Rey-Osterrieth Complex Figure - Immediate and Delayed Recall	Nonverbal learning and retrieval of complex visuospatial material.	Caffarra et al., [31], Carlesimo, [32], Shin, [34]
	Matched recall and recognition across modalities (e.g., Doors and People or equivalent)	Comparison of recall and recognition for verbal and visual material.	Smirni et al., [60], Warrington [61]
*Executive Functions and Cognitive Control*	Stroop Color-Word (or equivalent interference task)	Inhibitory control and resistance to interference.	Caffarra et al., [62]
	Modified Card Sorting Test	Abstraction, shifting, and perseveration.	Caffarra et al., [64], Nelson, [65]
*Patient-Reported Outcomes*	Depression inventory (e.g., BDI-II or another validated equivalent)	Severity of depressive symptoms.	Beck et al., [68], Ghisi, [69]
	Anxiety screening	Burden of anxiety symptoms.	Spitzer et al., [74]; Pietrabissa et al., [73]
	Epilepsy-specific quality of life (e.g., QOLIE-31)	Health-related quality of life: cognition, mood, medication effects, and overall impact.	Cramer et al., [77], Beghi et al. [76], Devinsky & Penry [78]

## Discussion

A structured sequence of neuropsychological assessment can clarify the cognitive and behavioral burden of epilepsy and translate test results into decisions that matter for patients [5]. Beyond seizure control, adults with epilepsy often need support in cognitive processing and self-regulation to maintain employment, social life, and autonomy [2, 3]. The proposed approach emphasizes measurement of core domains, careful attention to upstream constraints, and the routine inclusion of patient-reported outcomes.

Serial assessment is most informative when tied to a specific clinical question, such as a major change in seizure burden, a substantial medication adjustment, or another major management decision. When repeated testing is planned, clinicians should prefer instruments with alternative but comparable versions when available and interpret small score changes cautiously in light of practice effects. In clinical practice, neuropsychological data should inform, rather than replace, the broader risk-benefit discussion that includes seizure outcomes, safety, psychiatric status, and patient priorities [2, 3, 5, 6, 79, 80].

A first implication of this framework is that referral to neuropsychology should be question-driven rather than automatic. In newly referred patients, focused screening may be sufficient when the aim is to document baseline cognition, mood, and subjective complaints. By contrast, comprehensive assessment is warranted when the history suggests temporal lobe involvement, progressive functional decline, relevant psychiatric comorbidity, educational or occupational consequences, major antiseizure medication changes, or mismatch between patient complaints and bedside findings. This distinction helps balance clinical thoroughness with feasibility in resource-constrained services [4–6].

A second implication is that epilepsy should not be treated as a unitary cognitive syndrome. Even within the same broad diagnostic category, patients may differ substantially according to lesion type, seizure frequency, age at onset, developmental history, network organization, sleep disruption, antiseizure medication load, and psychiatric burden. Recent work on cognitive phenotyping and the International Classification of Cognitive Disorders in Epilepsy (IC-CoDE) reinforces this point by showing that adults with apparently similar temporal lobe epilepsies may present with distinct and clinically relevant cognitive profiles [8]. For this reason, a clinically useful battery must preserve cross-patient comparability while allowing syndrome-specific interpretation.

A third implication concerns differential diagnosis. Neuropsychological findings may raise suspicion that the clinical picture is being shaped by functional neurological symptoms, psychiatric distress, low effort, sleep disorder, or systemic factors, but they rarely provide a categorical distinction between epilepsy and its mimics. Controlled studies comparing epilepsy and PNES suggest that neuropsychological scores alone are not sufficient for diagnosis, although they may add incremental information when interpreted within a multidisciplinary formulation [10]. The main value of the assessment in these situations is often to clarify the relative contribution of objective impairment, emotional symptoms, health beliefs, and everyday disability.

A fourth implication relates to guidelines and international applicability. The proposed battery was tailored to Italian practice because availability of validated norms directly affects interpretability, especially for longitudinal decisions made at the individual level. Nevertheless, the clinical logic of the battery is not uniquely Italian. It is broadly consistent with ILAE recommendations that routine epilepsy care should include assessment of cognition, mood, and behavior, and that test selection must be driven by the referral question and local expertise rather than by a rigid universal template [5]. The Italian emphasis therefore concerns psychometric feasibility, not a different conceptual model of care.

Finally, network-based models of epilepsy, cognitive phenotyping, and precision medicine suggest that future batteries may become increasingly stratified. Rather than focusing only on lesion side or crude syndrome labels, the field is moving toward integrated profiles that combine neuropsychology with imaging, electrophysiology, genetics, and longitudinal outcomes. Our proposal does not attempt to operationalize a full precision-medicine framework, but it is compatible with that direction because it encourages modular assessment, careful syndrome-level interpretation, and repeated measurement when the clinical trajectory changes [2, 3, 8].

## Conclusions

Neuropsychological assessment is foundational to comprehensive adult epilepsy care. In non-surgical settings, its main value lies in clarifying who needs further evaluation, which domains are most relevant for a given syndrome or treatment phase, and how objective cognitive data can be integrated with psychiatric, electrophysiological, and imaging findings. The battery proposed here is intended as a structured and clinically feasible framework for Italian practice, aligned with international recommendations but adaptable to local resources and the individual referral question [4, 5]. A stronger evidence base will require further controlled studies comparing batteries, referral strategies, and longitudinal outcomes in defined epilepsy populations.

## Data Availability

No datasets were generated or analyzed for this article.
